# Spin states, vibrations and spin relaxation in molecular nanomagnets and spin qubits: a critical perspective

**DOI:** 10.1039/c7sc05464e

**Published:** 2018-03-07

**Authors:** Luis Escalera-Moreno, José J. Baldoví, Alejandro Gaita-Ariño, Eugenio Coronado

**Affiliations:** a Instituto de Ciencia Molecular (ICMol) , Univ. de Valencia , C/Catedrático Beltrán 2 , E-46980 Paterna , Spain . Email: alejandro.gaita@uv.es ; Email: eugenio.coronado@uv.es; b Max Planck Institute for the Structure and Dynamics of Matter , Luruper Chaussee 149 , D-22761 Hamburg , Germany

## Abstract

Very recently the closely related fields of molecular spin qubits, single ion magnets and single atom magnets have been shaken by unexpected results. We have witnessed a jump in the phase memory times of spin qubits from a few microseconds to almost a millisecond in a vanadium complex, magnetic hysteresis up to 60 K in a dysprosium-based magnetic molecule and magnetic memory up to 30 K in a holmium atom deposited on a surface. With single-molecule magnets being more than two decades old, this rapid improvement in the physical properties is surprising and its explanation deserves urgent attention. The general assumption of focusing uniquely on the energy barrier is clearly insufficient to model magnetic relaxation. Other factors, such as vibrations that couple to spin states, need to be taken into account. In fact, this coupling is currently recognised to be the key factor that accounts for the slow relaxation of magnetisation at higher temperatures. Herein we will present a critical perspective of the recent advances in molecular nanomagnetism towards the goal of integrating spin–phonon interactions into the current computational methodologies of spin relaxation. This presentation will be placed in the context of the well-known models developed in solid state physics, which, as we will explain, are severely limited for molecular systems.

## Abstract 2

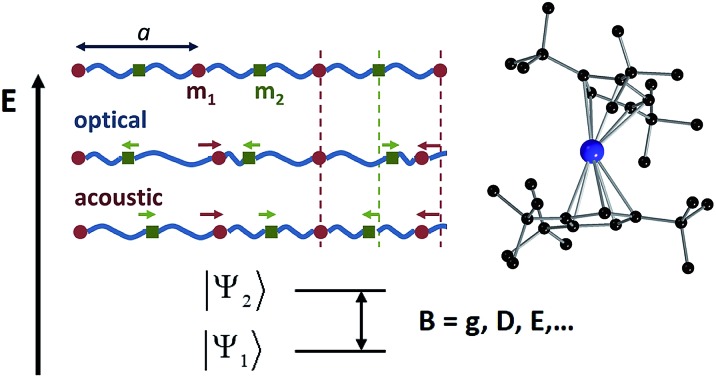
Spin–vibration coupling has been proven to be crucial for spin dynamics; theoretical studies are now addressing this experimental challenge.

## 1. Introduction

The understanding and control of spin dynamics at the nanoscale is an essential step towards the development of quantum technologies based on spin qubits. A quarter century ago, chemistry provided a unique testing bed to advance towards this goal in the form of molecular nanomagnets, which exhibit magnetic hysteresis at liquid-helium temperature.^1^,^2^ These molecules, characterised by a bistable magnetic ground state, have been proposed as promising candidates for information storage,^3^ magnetic refrigeration^4^ and several applications in molecular spintronics,^5^ nanotechnology^6^ and quantum computing.^7^–^10^ Unfortunately, low operating temperatures that are required for most of the reported entities to retain their magnetic bistability, and the rapid loss of quantum information, collectively known as decoherence, are two major obstacles that molecular nanomagnets still need to overcome for their practical implementation.^11^ This picture has only very recently started to change, based on the latest discoveries in three closely related subfields.^12^ In 2015, a molecular quantum two-level system (qubit) based on a vanadium(iv) trisdithiolate complex [V(C_8_S_8_)_3_]^2–^ displayed a record spin–spin relaxation time *T*_2_ = 670 μs,^9^ an order of magnitude above a record announced a few months earlier.^13^ The key to the success of [V(C_8_S_8_)_3_]^2–^ was fine property optimization: the use of a planar rigid ligand that is free of nuclear spins and at the same time affords solubility in CS_2_, a heavy solvent that is also free of nuclear spins. However, *T*_1_ displays a strong thermal evolution, eventually limiting the coherence time at higher temperatures. A year later, the very first single-atom magnet, in the form of a single Ho atom adsorbed on a magnesium oxide film grown on a silver substrate, showed magnetic memory up to 30 K and bistability that lasts for 1500 s at 10 K.^14^ The very low phonon density of MgO that plays a very dominant role at low temperature was shown to be critical and served to insulate the Ho atom from the soft phonons on the Ag substrate. In 2017 yet another record was shattered with the discovery of magnetic hysteresis on a mononuclear dysprosium complex based on optimized arene ligands at an extraordinarily high temperature of 60 K by two independent studies.^15^,^16^ Additionally, hysteresis at high temperatures has recently been achieved by using very fast sweep rates, namely 30 K at 200 Oersted per second.^17^,^18^ Such a rapid enhancement of properties opens new perspectives in molecular magnetism and demands urgent attention.^19^

These experimental records have been supported by advances in theoretical modelling, but a precise description of spin dynamics at the nanoscale is still extremely challenging. For many years, modelling of slow magnetic dynamics in nano-objects, such as single-molecule magnets and single-ion magnets, relied mostly on the Orbach mechanism. The effective barrier for the reversal of magnetization is now routinely estimated from first principles,^20^,^21^ which allows a rational design of these nanomagnets.^22^–^24^ In contrast, Raman processes are very often taken into account only parametrically. This evidences that control over spin dynamics in molecular nanomagnets is still an open problem, which requires the modelling of vibrations and of their coupling to the spin energy levels from first principles.

Herein, we discuss the current difficulties in the search for a relationship between the molecular structure and spin dynamics. In order to get an appropriate perspective, we will start from the achievements and drawbacks of a static picture that aims to correlate chemical structures with the spectroscopic and magnetic properties of molecular nanomagnets; then, we will pass through the problematic focus on what we argue is the first stage of this problem – the energy barrier – and, finally, we will review what is being nowadays recognised as the current stage of this problem: the role of vibrations.

## 2. Magnetic energy levels in the static picture: a controversial barrier

**Fig. 1:**
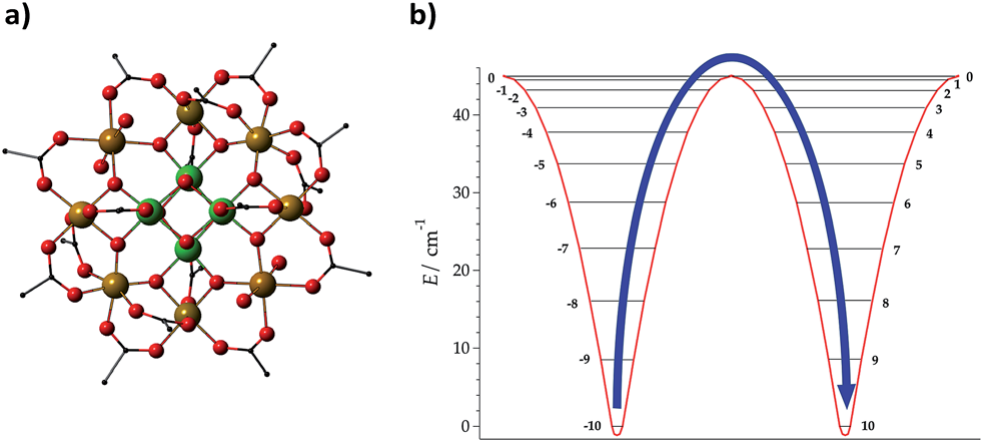
Molecular structure of (a) Mn_12_O_12_ (CH_3_COO)_16_(H_2_O)_4_, denoted as Mn_12_, and (b) energies of different *M*_S_ projections in the ground spin multiplet of Mn_12_; an effective energy barrier *U*_eff_ = |*D*|*S*^2^ for spin inversion between *M*_S_ = +10 and *M*_S_ = –10 is derived from the spin Hamiltonian *H* = *DS*_*z*_^2^. Mn: yellow + green, O: red, and C: black; H is omitted for clarity.

The development of a theoretical framework intended to provide an accurate description of experimental observations has been considered as a first milestone in the field.^25^ The assumption of an Orbach mechanism and thus the consideration of the effective barrier as a key factor for the slow reversal of magnetisation started with the first generation of molecular nanomagnets, based on the dodecanuclear manganese cluster Mn_12_O_12_(CH_3_COO)_16_(H_2_O)_4_, which was called the *Drosophila melanogaster* of single-molecule magnets (SMMs) (see Fig. 1(a)).^1^ For these polynuclear transition metal complexes with SMM behaviour, the efforts for a rational design were mainly focused on the optimisation of a large ground state spin, *S*, and a large negative zero field splitting parameter, *D*, to increase the energy barrier through the relation *U* = |*D*|*S*^2^ (Fig. 1(b)).^26^,^27^

In contrast, the magnetic properties of SIMs and mononuclear spin qubits are largely determined by the magnetic anisotropy of a single ion, which results from the combination of spin–orbit coupling and the crystal field.^28^ The relative strength of such electronic interactions relies on the electronic configuration of the magnetic centre, with remarkable differences between d-block (ligand field > spin–orbit coupling) and f-block element ions (ligand field < spin–orbit coupling).^29^ In the latter we can also distinguish between lanthanides and actinides.

Crystal field theory is key for the description of the energy level scheme. This frequently requires the determination of a large number – up to 27 – of crystal field parameters (CFPs). The estimation of CFPs can be done based on a few alternatives. The first one is the rationalisation of the experimental features of complexes already synthesised and characterised empirically. This has traditionally been the default option of spectroscopists and consists in the direct fit of spectroscopic data while varying a non-negligible set of CFPs. The non-vanishing CFPs depend on the point group of symmetry of the molecule.^30^ An accurate description of magnetic properties following a phenomenological approach has also included thermodynamic techniques, such as powder and single-crystal magnetic susceptibility and torque magnetometry.^31^ The second option is using a computational approach to obtain CFPs and then modelling magnetic properties, or even to predict them using the real chemical structure of the coordination complex as an input. In this direction, there are mainly two alternatives that have proven to be useful in molecular magnetism, namely the electrostatic crystal-field approach, which considers a point-charge distribution around the central ion,^32^–^35^ and the more expensive *ab initio* calculation, based on the complete active space self-consistent field (CASSCF).^36^–^40^ Comparisons between these two approaches have been made elsewhere.^41^,^42^

Partly guided by theoretical efforts, a rich community of experimental chemists and physicists has worked for decades to increase the energy barrier with the objective of achieving high blocking temperatures, ideally up to room temperature. Popular chemical families include beta-diketonates, aromatic rings, polyoxometalates and phthalocyaninato anions as ligands, and the consensus seems to favour Kramers ions with an oblate f-shell distribution (and notably the Dy^3+^ ion), with an axially elongated coordination environment, rigid polyhapto ligands and diamagnetically diluted samples. There are now record barriers (assuming an Orbach mechanism) that are at least an order of magnitude higher than those reported in cluster-type SMMs. At the same time, SIMs working at room temperature are still a distant dream. One of the main reasons is that the employed energy-barrier framework is an oversimplification. Thus, while recognizing the important victory of being able to systematically design and prepare systems with higher effective thermal barriers, we need to put this into perspective.

Three figures of merit have been frequently employed in the analysis of the dynamical magnetic properties of SMMs:

(1) The effective barrier *U*_eff_, which can be quantified by fitting the variation of the ac susceptibility signal with frequency and temperature to the Arrhenius equation.

(2) The first excited magnetic energy level *E*_1_, which is ideally determined by spectroscopy, but frequently just estimated by theoretical calculations.

(3) Hysteresis loops, which can be observed below a certain blocking temperature. This is actually the critical parameter that bars the gate for devices and applications, although in the field it is common to see “blocking temperature” used in relation to the ac magnetometry signal, since not all complexes display hysteresis at 2 K.

**Fig. 2:**
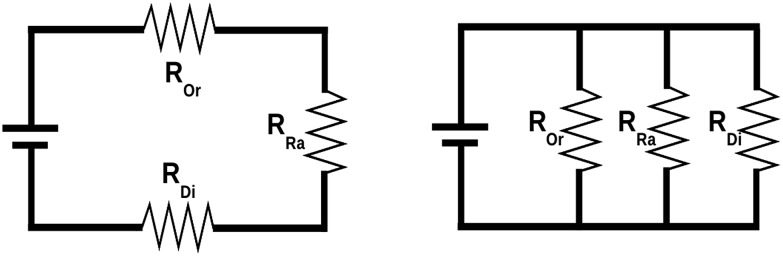
Series circuit (left) *versus* parallel circuit (right). Modified with permission from Mets501 (CC by-sa 3.0) series circuit and parallel circuit.

It has often been assumed that *U*_eff_ and *E*_1_ are identical and directly control the blocking temperature. However, individual studies have repeatedly shown that this is not the case,^43^ with *U*_eff_ being significantly lower than *E*_1_ (which has recently been attributed to the presence of off-resonance phonons due to the finite phonon lifetimes which offer a wider energy window),^44^ or with both *U*_eff_ and *E*_1_ being two orders of magnitude higher than the blocking temperature.^45^ What is happening? It is likely that there is no single answer, but it seems clear that all relevant physical processes – including Orbach (Or), Raman (Ra) and direct (Di) mechanisms – should be taken into account in each case. This is conceptually not so different from a simple electric problem, which we shall use for illustration purposes. Let us picture two electric circuits (see Fig. 2), one in series and the other in parallel, for which we want to minimise the current flow, just like we want to minimise spin relaxation in our molecular magnets. What is the simplest systematic strategy to increase the overall resistance in a simple circuit? In the series circuit, we can just pick any resistor, say *R*_Or_, and raise its resistance, and *R*_series_ will escalate with no limit. In the parallel circuit the situation is different: when *R*_Or_ rises over a certain threshold the current flows exclusively through *R*_Ra_ and *R*_Di_—the paths of least resistance—making *R*_Or_ an irrelevant part of the circuit.

Back to molecular magnetism, we qualitatively have a similar situation: given several relaxation pathways, the spin will most commonly relax *via* the fastest one. It is therefore easy to understand that, after the thermal barrier has risen over a certain threshold, the spin will just use a different relaxation mechanism. So, further raising the barrier and thus blocking the path of most resistance will be irrelevant for all practical purposes. In the case of SIMs, the community has already done a good job in raising the barrier and is now starting to admit that molecular vibrations are the next pathway that needs to be blocked. Actually, Liddle and van Slageren, in a tutorial review published in 2015, already pointed out that the magnitude of the crystal field splitting is not the only factor governing the slow relaxation of molecular nanomagnets. They highlighted the importance of Raman processes and explicitly indicated the necessity, for energy dissipation, of transferring the energy, *via* phonons, from the spin system to the thermal bath.^29^ For the study of these phenomena, we need to take advantage of the tools, methods and concepts that were developed by physicists working on the thermal dependence of the crystal field Hamiltonian. Thus, we will now overview such reports, summarizing the most crucial equations that relate vibrations and spin energy levels, as well as the open problems that still need to be addressed.

## 3. Spin relaxation and vibrations

The tools for the study of spin–vibration coupling were originally developed to determine the thermal and vibrational modulation of spin energy levels in simple solids. To understand why this framework has limited applicability in the case of molecular solids, and thus why new approaches are being developed, we will start by a brief historical tutorial review, including a summary of the key approximation and results. Readers interested in the procedures that are currently being proposed to guide the chemical design may want to go directly to the subsections: *An improved, plastic, new general methodology* and *Chemical strategy and theoretical perspective*.

### Historical contributions to the electron–phonon interaction

In 1969, Shrivastava showed for the first time that the thermal dependence of spin energy levels, in particular zero-field splitting (ZFS), cannot always be described by the static modulation of spin energy levels *via* lattice thermal expansion. Instead, sometimes one needs to consider a dynamic effect caused by Electron–Phonon Interactions (EPIs), where both acoustic and optical phonons‡
‡Phonons are lattice vibrations that can be imagined as particles carrying a quantum of vibrational energy. can be involved.^46^–^48^ Soon, the need to rationalise the thermal evolution of other magnetic anisotropy parameters in terms of their static and dynamic (EPI) constituents,^49^,^50^ especially at high temperature, was evidenced. Related studies of the effect of localised modes on the thermal dependency of the spin–lattice relaxation time *T*_1_ are even more closely connected with our current focus. In some systems it was found that a picture consisting only of delocalised lattice vibrations was not enough to explain certain experimental results.^51^,^52^ These theoretical advances introducing the EPI constituted a new paradigm that was adequately able to describe the thermal dependence of spin energies,^53^ even beyond the long wavelength approximation (LWA, see below).^54^,^55^

### Step by step scheme, approximations and limitations

Let us review the historical scheme by commenting on the limitations introduced by each of its approximations. In a nutshell, there are three steps to obtain the thermal dependence of spin energy levels, or, in general, the relevant parameter *B* to be studied, such as the ZFS, *D*, or the Landé factor, *g*:

(i) The calculation of the static contribution *B*_stat_

(ii) The use of the Debye model to obtain the contribution *B*_ac_ of the acoustic branches of the phonon spectrum

(iii) The approximation of optical branches using a single-phonon model to obtain their collective contribution *B*_op_.

Following this scheme, the thermal dependence *B*(*T*) is decomposed into three terms:

1 *B*(*T*) = *B*_stat_(*T*) + *B*_ac_(*T*) + *B*_op_(*T*)

where *T* is the temperature, *B*_stat_ denotes the static modulation, and *B*_ac_ and *B*_op_ account for the acoustic and optical phonon contributions of the dynamic term or EPI, respectively.

Regarding step (i), the calculation of the static contribution was generally achieved either by means of thermal expansion coefficients—either by a complete diagonalisation or by using perturbation formulae—or by using point-charge models.

**Fig. 3:**
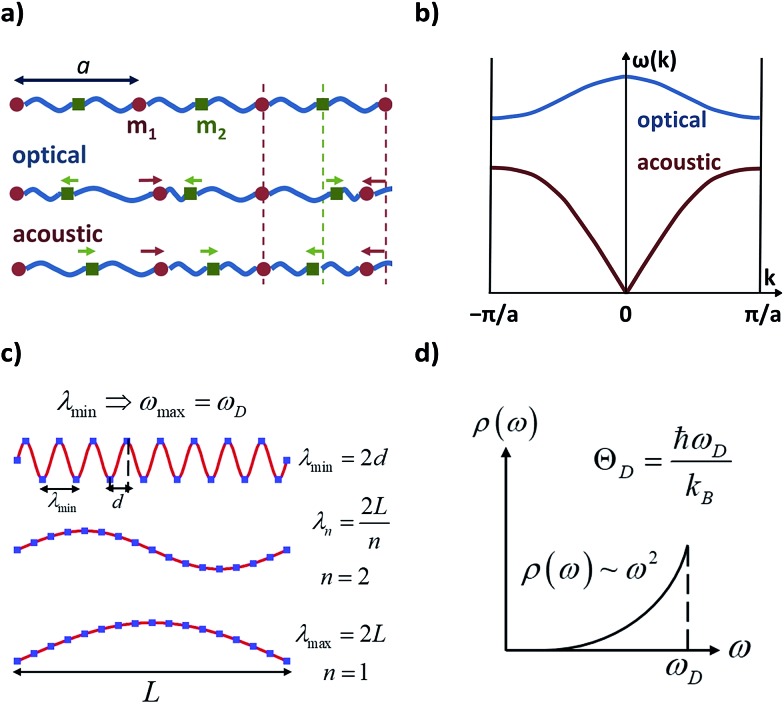
Characteristic features of acoustic and optical phonons in a solid and the Debye model. Reused with permission from Brews ohare (CC by-sa 3.0) diatomic phonons and optical & acoustic vibrations. (a) Linear diatomic solid with “*a*” being the lattice parameter; acoustic and optical phonons assimilated as in-phase and out-of-phase movements. (b) Acoustic and optical branches (dispersion relations) as a function of wavenumber *k* in a linear diatomic solid. (c) Transverse vibrations in a 3D solid; the atomic separation *d* imposes a minimum wavelength, and thus, a maximum frequency, *ω*_D_ (Debye frequency). (d) Density of phonons *ρ* available at each frequency as given in the Debye model; *ω*_D_ can be translated in terms of the Debye temperature, *Θ*_D_.

The following step (ii) is central to our interests and relies on the Debye model (explained in Fig. 3), which is known for being the first approach to correctly reproduce the behaviour of the specific heat in simple solids. Within this model, one starts with the generalised coordinates that describe each atomic motion in a solid according to a given phonon *k*:^50^
2

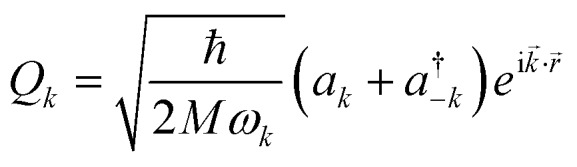

where *M* is the mass of the crystal, *ω*_*k*_ is the phonon frequency with *k* as the wave vector, *a*_*k*_ and 
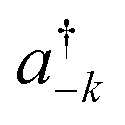
 are the phonon operators, and *r* represents the atomic locations.

The main difficulty in eqn (2) usually comes from the evaluation of the exponential factor. An approximated solution is possible by combining the assumption of cubic symmetry with the so-called long wavelength approximation (*i.e.* |*k·* *r*| ≪ 1). Thus, the phase factor *e*^i*k*^*^·^*^*r*^ can be approximated as *k·* *r*. Here one finds the first remarkable limitation for our purposes, as complex molecular crystals generally lack cubic symmetry. Moreover, systems where the LWA fails can be encountered in the literature, especially when the working temperature is of the order of the Debye temperature or higher.^55^ Indeed, as the temperature is raised, phonons of short wavelength are also excited and thus the integral in eqn (4) cannot properly describe a correct temperature dependence. In the context of molecular crystals, which present Debye temperatures of the order of tens of kelvin,^56^ this failure is expected to appear much below room temperature.

An expansion of *B*_ac_ and *B*_op_ in terms of these coordinates gives rise to expressions that depend on expectation values 
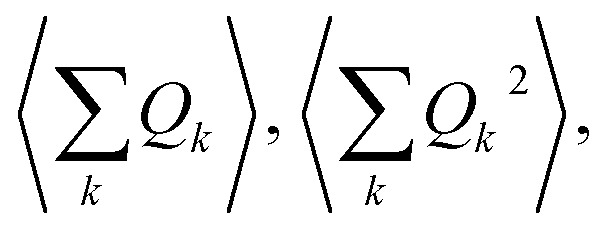
 and so on. Generally, under an anharmonic phonon model for atomic displacements, 
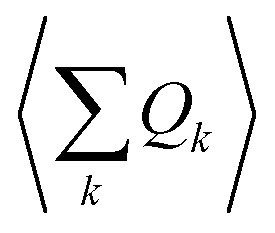
 may be non-zero. In contrast, if the model is harmonic, this expectation value is identically zero, while 
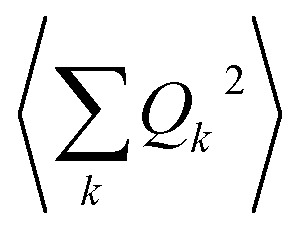
 would be the first term in the expansion different from zero. The expressions derived by Shrivastava are truncated at second order and consider harmonic phonons. Thus, only the effect from quadratic atomic displacements is incorporated,^50^,^57^ and the phonon-induced modulation of *B*_ac_ is proportional to 
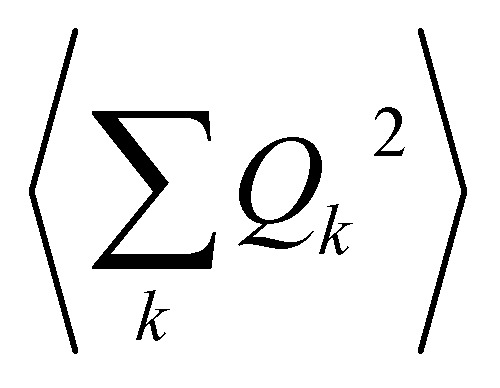
. Whereas this approach has also been successfully recovered by the models recently proposed for magnetic molecular crystals,^48^ we need to point out that there is a second important limitation. There are relevant anharmonic effects, such as lattice spacing or phonon–phonon interactions, especially at high temperatures, which cannot always be safely ignored.^60^ Recently, the relevance of anharmonicity in phonons for spin dynamics has already been the subject of study in the context of magnetic molecules.^44^

As phonon energies are close enough to describe a continuum, the series 
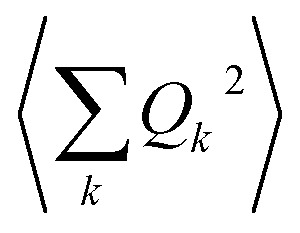
 is usually converted into an integral, which describes the overall contribution of the acoustic phonon spectrum to *B* for harmonic quadratic atomic displacements. This integral introduces a third limitation: it impedes determining which phonon modes in 
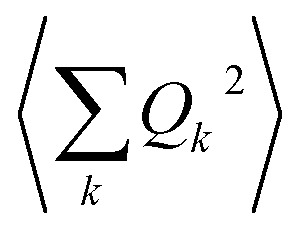
 contribute the most to modulate *B*_ac_. Since one of the main relaxation pathways in molecular spin qubits and SMMs can be *via* spin–vibration coupling, it would be desirable to be able to check each individual mode contribution in order to rationally design these molecular systems and slow down this relaxation. Fortunately, this is trivial to do, simply by keeping the series expression instead of switching to the integral.

Before 
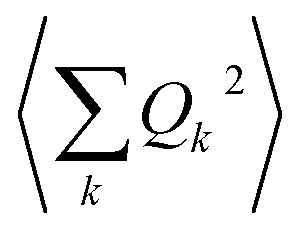
 is converted into an integral, the sum over the square atomic locations involved in each collective motion 
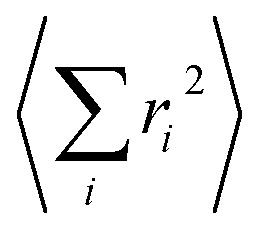
 is substituted by a mean value *R*^2^, with *R* being the lattice nearest-neighbour distance in the considered crystal of cubic symmetry. The conversion of the series into an integral then gives:
3

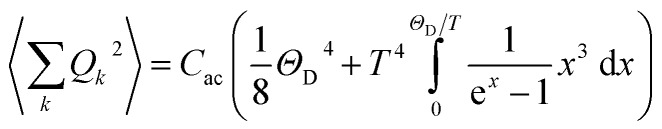

with 
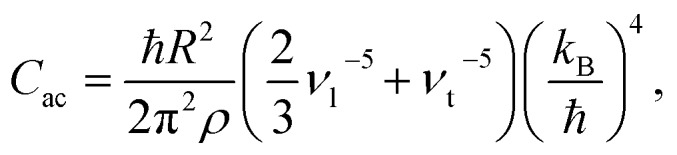
 *ρ* the crystal density, *v*_l_ and *v*_t_ the longitudinal and transverse sound velocities in the crystal, and *Θ*_D_ the Debye cut-off temperature.§
§Using the definition of the Debye temperature (see Fig. 3), *k*_B_*Θ*_D_ is equal to the phonon energy of maximum frequency. Thus, the Debye temperature can be interpreted as the temperature at which the highest-frequency vibration (hence, every one of them) is excited. Macroscopically, the Debye temperature can be regarded as a measure of the hardness of the crystal. Typical Debye temperatures range from 38 K for cesium to 2230 K for carbon. Longitudinal and transverse velocities can be rewritten in terms of an effective sound velocity *v*: (2/3)*v*_l_^–5^ + *v*_t_^–5^ = 3*v*^–5^.¶
¶Let us picture a vibration that propagates in the direction that is perpendicular to a given crystallographic plane. This will be a longitudinal phonon if the stretching and compression happen between successive planes, so that the geometric distortion is parallel to the direction of propagation. Conversely, it will be a transverse phonon if there is a lateral displacement between successive planes, so that the geometric distortion is perpendicular to the direction of propagation. In a one-dimensional solid, atoms are restricted to move along a given straight line, so phonons corresponded to longitudinal waves. In three-dimensional solids, atoms are not restricted anymore to the direction of propagation, and can also vibrate up and down, producing transverse waves. An effective sound velocity is commonly used to describe the speed propagation of a phonon, where one can distinguish a longitudinal and a transverse velocity, respectively. This effective sound velocity is also related to the hardness of the crystal. Hence, the thermal dependence of *B* due to acoustic phonons *B*_ac_(*T*) is given by:^60^,^61^

4

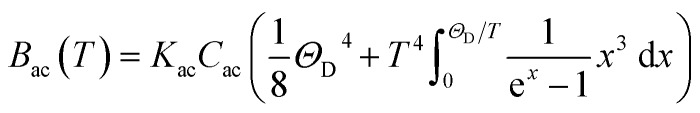

where *K*_ac_ is the proportionality constant between *B*_ac_(*T*) and 
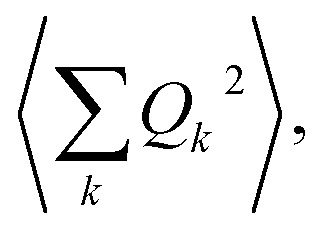
 and depends on the spin–phonon coupling strength. The factor *K*_ac_*C*_ac_ is usually taken as an adjustable parameter and the term *B*_ac_(0) = *K*_ac_*C*_ac_(1/8)*Θ*_D_^4^ is called the zero-point acoustic phonon contribution.

The Debye model presents practical and fundamental limitations. Experimentally determined values of *Θ*_D_ sometimes differ by tens or even hundreds of Kelvin depending on the technique.^62^–^64^ In other cases, the thermal dependence of *Θ*_D_ in eqn (4) is employed as a last resource to fit room-temperature data.^64^–^66^ It may work and provide practical applications,^60^ but makes *Θ*_D_ unphysical since eqn (4) should only be used as long as the LWA is fulfilled, *i.e.*, at not too high temperatures.

Note that the Debye model is useful for simple solids but has a limited applicability in molecular solids. First, this model assumes a specific phonon spectrum, which could fail in complex crystals of a rather general symmetry where the paramagnetic entities cannot be considered zero-dimensional anymore. Second, the dispersion relation *ω* = *v·k* has been used to express the integral in eqn (4). Although this relation is frequently employed, it might not work for some systems and should be consequently replaced by another one depending on the specific structure and properties of the crystal. Third, there is a well-known danger of using the Debye model at high temperatures,^46^ which is now the most interesting regime for the communities of molecular magnetism and spin qubits.^15^,^67^,^68^ Already in 1969 it was claimed that theoretically probing the high temperature regime would only be possible whenever *non-Debye calculations* were available, which should be *point-to-point calculations*.^46^ These calculations should consider explicitly the exact phonon spectrum of each particular crystal, and possible angular^54^ and thermal dependencies of sound velocities in a given crystal. Others have elaborated on this point, discussing about the replacement of the Debye phonon density by the real one.^52^,^64^,^66^ Over the last few years, it has been pointed out that fitting temperature dependencies of spin–lattice relaxation times sometimes requires using the real phonon density.^69^,^70^

Finally, in step (iii), the contribution of the optical branches is accounted for by all the optical modes:
5

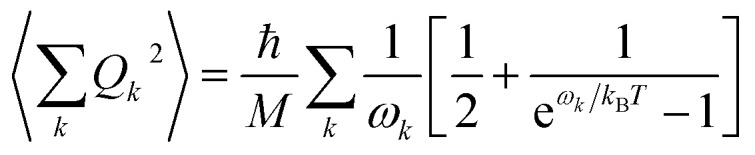




The phonon optical branch is described using a single-mode harmonic model, with effective frequency *ω*_eff_ and distortion coordinate *Q*. This constitutes the fourth main limitation in this procedure, which is usually justified by stating that optical phonons have been usually found in narrow frequency ranges. In the case of molecular solids, this is no longer the case, since the frequencies of molecular vibrations span over two orders of magnitude.

The calculation of *Q*^2^ results in:
6

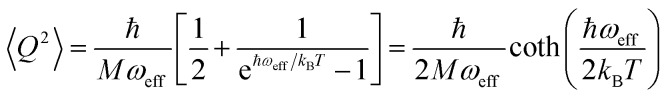

and, the thermal dependence of *B* due to optical phonons *B*_op_(*T*) is obtained as:^71^
7

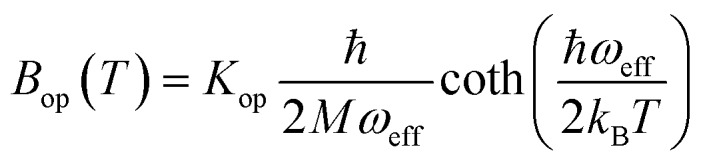

with *K*_op_ now being the proportionality constant between *B*_op_(*T*) and *Q*^2^ characterizing the strength of the spin–phonon interaction. The factor 
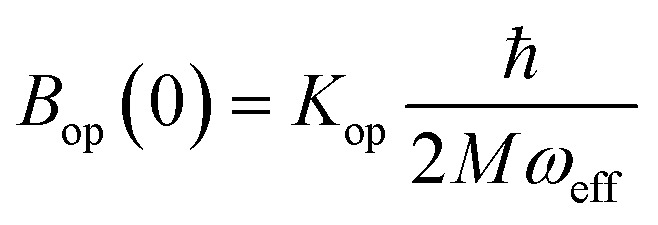
 is the zero-point optical phonon contribution and is also usually taken as an adjustable parameter.

Even with these four main limitations, this procedure has produced useful insights into the theoretical rationalization of spin dynamics in magnetic molecules. For illustration, we can comment on an electron spin relaxation study by Eaton and co-workers that considered a series of Cu(ii) complexes in a wide temperature window.^72^ The *semi-empirical* model they used invoked contributions from several relaxation processes achieving an excellent reproduction of the thermal dependence of the spin–lattice relaxation time, *T*_1_. An almost temperature-independent direct process was found to be significant below 20 K, Raman processes dominated between 20 K and 60 K, and local modes of energies around 300 K (200 cm^–1^) were found to be very significant already at temperatures of 60 K and above. Since no low-lying electronic states are expected for Cu(ii) complexes, these authors did not even consider Orbach processes, in contrast to the previously mentioned excessive focus on the barrier that has been so pervasive in the SMM community.

The same authors demonstrated how detailed experimental information can be useful to find the correct relaxation mechanism. In the case of bis(diethyldithiocarbamato) copper(ii), Cu(dtc)_2_ (chemically diluted into a diamagnetic analogue), *T*_1_ was found to be frequency independent. This ruled out a mechanism involving a thermally activated process and instead indicated that relaxation proceeds *via* a local mode. In general, distinguishing between a local mode and a thermally activated process requires experimental data at temperatures up to or beyond the temperature corresponding to this characteristic energy or relaxation measurements with at least two different microwave frequencies.

Besides all the above mentioned limitations, this whole methodology is *semi-empirical*.^57^,^63^,^73^–^77^ The relevant parameters are extracted by means of fittings to experimental data, from independent experiments or tabulated values, or simply estimated. Without an independent predictive capability, this means that these models cannot facilitate a rational molecular design.

### An improved, plastic, new general methodology

**Fig. 4:**
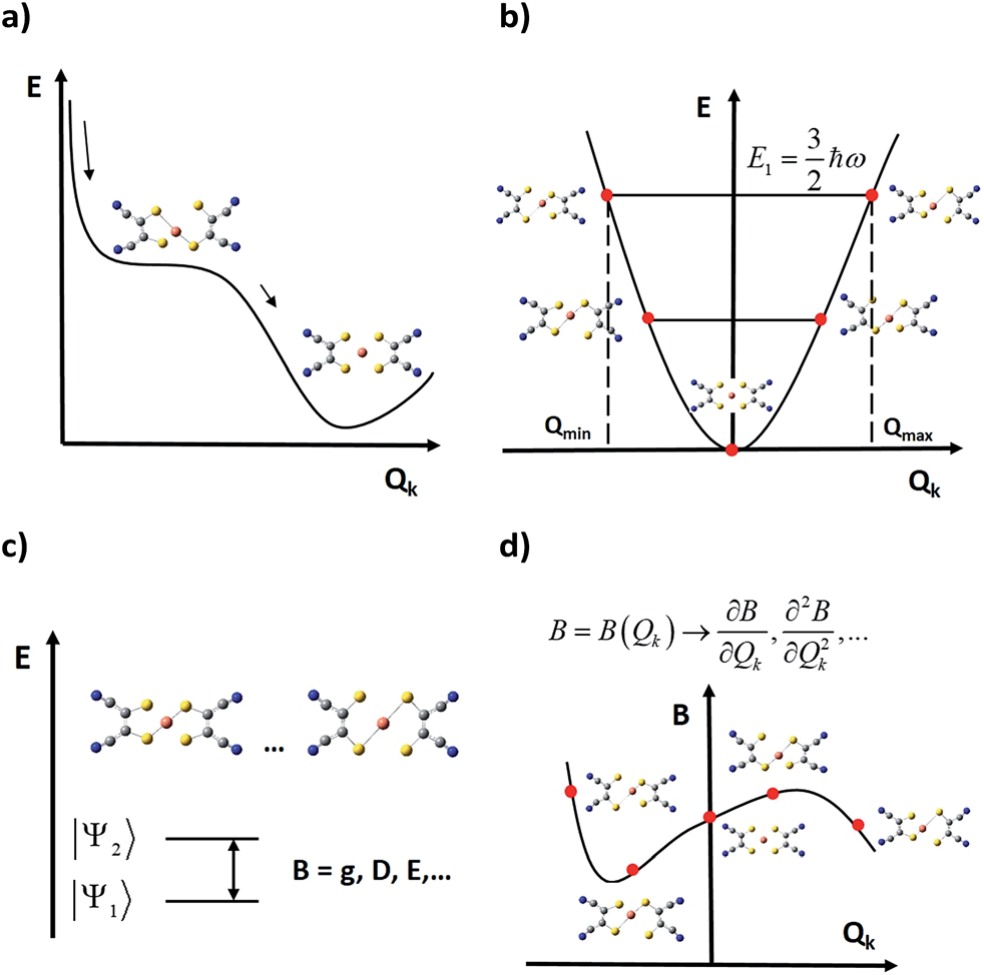
Schematic representation of current general methodologies for studying the effect of vibrations on the spin relaxation; *E* is the energy and *Q*_*k*_ is the normal (distortion) coordinate of a given normal (vibrational) mode. (a) Calculation of the minimum potential energy geometry and its vibrational spectrum. (b) Generation of distorted geometries around the equilibrium geometry following each mode. (c) Computation of relevant parameters *B* at each distorted geometry. (d) Extraction of relevant quantities to be used in a master equation from the vibrational modulations of *B*.

As spin–phonon coupling depends on the fine details of each particular lattice, to capture the wide spectrum of molecular crystals any theoretical model should be *plastic* enough to incorporate all system subtleties. This means distinguishing and studying the effect of each individual vibrational mode, and this is the starting idea which current models rely on, from 2015^58^,^59^ and henceforth.^15^,^44^,^78^ Broadly, the general method consists of four main steps (see Fig. 4):

(i) One starts by relaxing the relevant geometry and calculates its vibrational spectrum. Depending on the case, this geometry may only involve atoms of the magnetic complex or, additionally, atoms of the rest of the unit cell. This is commonly undertaken *via* DFT (Density Functional Theory), although recent approaches based on general force fields are also available.^79^ However, DFT does not necessarily guarantee a decrease in the overall system energy as the atomic orbital basis is enlarged. Thus, depending on previous experience, finding a systematic method to relax the geometry may become a hard task. In this context, there are three methodological issues that need to be discussed. First, one should not guide the relaxation process aiming for a perfect match between the X-ray structure—usually extracted at *T* ≥ 100 K—and the relaxed geometry, which is at the absolute energy minimum of the chosen theoretical method.^29^,^48^,^67^ This can be solved either by low-temperature crystallography or by correcting high temperature effects such as libration in the ≥100 K experimental geometry. Second, if the steric pressure by the environment is crucial for the molecular structure, it needs to be taken into account by including, for example, a set of frozen nearest counter-ions during the geometrical optimisation.^48^ Only occasionally can this be dropped and perform a relaxation in a vacuum. Third, usually the calculation of phonons is computationally demanding; thus, only one or a few directions in the reciprocal space are chosen, which limits the physical value of the results. For instance, if only the unit cell gamma-point is taken into account, only vibrational modes restricted to a single unit cell can be taken into account, meaning that the vast majority of intermolecular modes are neglected.^44^,^78^ Note that the more modes are included in the model, the more likely it will be to find all the potential spin relaxation channels. Whenever the number of modes becomes too large, one will have to sense and select only those ones that could contribute the most to relaxation.

(ii) In a second step, the relaxed geometry is distorted following each vibrational mode, generating a finite set of distorted geometries. For each mode, the selection of the lower and upper bounds of the distortion coordinate is not unique, but one criterion may be distorting the geometry until reaching the energy of the first excited vibrational state. Thus, one can safely use the energies of a harmonic oscillator. Likewise, the criterion used to choose those discrete values that each distortion coordinate must take is not unique yet either.^33^,^48^

(iii) Once the relevant magnetic anisotropy parameters affecting the spin relaxation are identified, they can be extracted from each distorted geometry by means of either *ab initio* or DFT point calculations. For instance, among these parameters one can find the *g* factor in spin–1/2 molecular spin qubits,^48^ or crystal field parameters such as the ZFS.^15^,^44^,^78^ A critical approximation at this step arises when periodic boundary conditions are not incorporated into these point calculations, so they are performed on a single isolated molecule. This may impose a severe limitation on the calculation quality, as long-range effects derived from the presence of charged species in the crystal are being completely removed. One can partially overcome this problem by placing near point charges simulating the outer electrostatic shells of the molecule and testing whether these effects are important or not.^44^

(iv) Finally, the spin–vibration coupling is introduced as the modulation that each vibrational coordinate exerts on the relevant parameters. This coupling is characterised *via* derivatives of these parameters with respect to the vibrational coordinates, which are calculated either analytically or numerically and employing the results of the above point calculations. At this point, it is still important to develop and agree on a robust procedure to calculate these derivatives. This can be an important source of numerical error depending on their quality.^33^,^48^,^67^ The remarkable achievement of this methodology is that assumptions derived from the Debye model are no longer required as spin–phonon coupling coefficients are individually and explicitly evaluated.

The last stage that completes this process and connects with measurable magnitudes like magnetic relaxation times or spin decoherence times is the inclusion of the calculated spin–phonon coefficients in an appropriate master equation.

### Chemical strategy and theoretical perspective

Let us now briefly revise the experimental examples that, as mentioned above, have shaken this research field. We shall start with the several small coordination complexes based on transition metals and rigid polyhapto ligand ions that have been recently highlighted because of their long relaxation times.

**Fig. 5:**
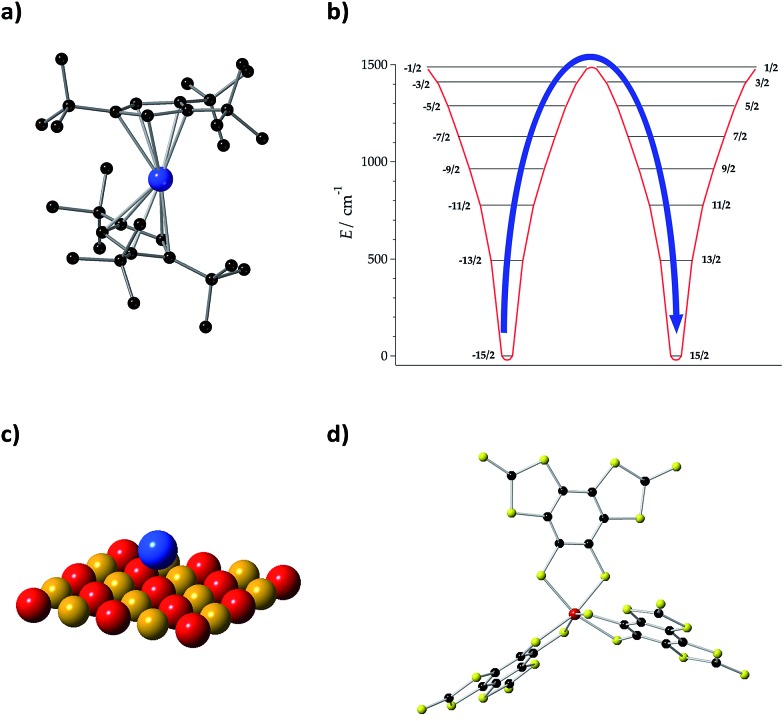
(a) Molecular structure of [Dy(Cp^ttt^)_2_]^+^, where Cp^ttt^ = {C_5_H_2_^*t*^Bu_3_-1,2,4} and ^*t*^Bu=C(CH_3_)_3_. H atoms are omitted for clarity. Dy: blue and C: black. (b) Energy levels and *M*_*J*_ projections of [Dy(Cp^ttt^)_2_]^+^, determined by CASSCF-SO calculations. (c) Ho atom on a MgO monolayer. Mg: yellow and O: red. (d) Molecular structure of [V(C_8_S_8_)_3_]^2–^. V: red, C: black, and S: yellow.

Initially, we need to focus on the record value for the spin–spin relaxation time *T*_2_ = 675 μs (at *T* = 10 K) which was set in 2015 by the vanadium complex [V(C_8_S_8_)_3_]^2–^ (Fig. 5(d)) with perdeuterated tetraphenylphosphonium counter-ions and in a diluted frozen CS_2_ solution, avoiding nuclear spins.^9^ At low temperature, *T*_2_ is governed by temperature-independent interactions with the spin bath, which in this case is unusually low. However, as the number of active phonons increases with rising temperature, *T*_1_ decreases and eventually limits *T*_2_. In this case, *T*_2_ decreases by about an order of magnitude for every rise in temperature of 30–40 K, signalling a strong coupling between spin states and vibrations.

In the opposite extreme, one finds vanadyl phthalocyanine VOPc,^68^ with an almost constant *T*_2_ = 1 μs between 5 K and 300 K (as usual, at high dilutions). For VOPc, a preliminary analysis attributed the high values of *T*_1_ and *T*_2_ at high temperatures to the rigidity of the vanadyl moiety, and such rigidity was also shown to be important in another related study.^67^ If the V–O vibration is the only one that couples with the spin state, its frequency would govern the temperature at which *T*_1_ starts to be short. It would be tempting to speculate on whether the marked difference between [V(C_8_S_8_)_3_]^2–^ and VOPc in the thermal dependence of *T*_1_ is related to the vanadyl moiety or to their very different environments—a crystal compared with a frozen solution—but since this is a multifactorial problem, calculations are required before jumping to conclusions.

Another well-studied case in this series is [Cu(mnt)_2_]^2–^,^13^,^48^ which displays *T*_2_ = 68 μs at low temperature and *T*_2_ = 600 ns at 300 K. We identified the two modes with the strongest spin–vibration coupling in the range 100–300 K; these involve distortions outside the molecular plane.^59^ In fact, this molecule is planar in the crystal, but theoretically relaxes in a vacuum to a skewed structure. One can extrapolate that increasing the chemical pressure driving the planarity of the complex will raise the vibrational frequency of these modes, thus decreasing their availability as spin relaxation paths. This could result in the survival up to higher temperatures of long spin–lattice relaxation times *T*_1_, and, indirectly, in long spin–spin decoherence times *T*_2_ up to higher temperatures. In contrast, frozen solution samples permit *T*_1_ measurements in the absence of crystalline pressure and, indeed, these measurements revealed shorter *T*_1_ times for this complex.^80^

Lattice vibrations were equally critical in explaining the behavior of dysprosocenium,^15^ a record-setting single-ion magnet based on a single Dy(iii) cation sandwiched between two (1,2,3)tri-*tert*-butyl pentacene anions (Fig. 5(a)). This system presents a crystal field splitting of about 1500 cm^–1^ (Fig. 5(b)), magnetic hysteresis at temperatures of up to 60 K and an effective barrier *U*_eff_ = 1223 cm^–1^ (1760 K), something that could naïvely be identified with an extremely uniaxial coordination environment in an ideal geometry. In fact, this was claimed in a parallel discovery of the same record SIM.^16^ This claim does not correspond to the reality of the molecular structure: the complex has a bent shape and bears no correspondence with any ideal symmetry, pseudoaxial or not. Instead, its unique spin dynamics were related to an equally unique spin–phonon coupling of the constrained metal–ligand vibrational modes, intrinsic to the bis-η^5^-Cp^ttt^ coordination geometry. In particular, four modes have been identified as detrimental, in the sense of coupling strongly to spin states that participate in spin relaxation. These modes involve motion of the two C–H groups on each aromatic ring. This moved the authors to suggest the substitution of these groups by heavier analogues. In cases like this, deuteration would have a minor practical effect compared with halogenation or substitution by an organic group R, but at the same time it would allow a cleaner theoretical analysis since the static crystal field effect would be essentially intact.

Let us finally focus on single atom magnets, in which a neutral atom sits on the top of an insulator. In the first studied example, the neutral atom is Ho and a thin MgO layer separates it from an Ag substrate (Fig. 5(c)).^14^ When physicists described this minimalistic system they highlighted the role of the MgO layer as “protecting the quantum magnet from scattering with electrons and phonons of the substrate” or, in other words, decoupling it from the phonon and conduction electron baths. In this case, instead of blocking the detrimental modes that couple with the electron spin as we suggest in the chemical approach, this is achieved by choosing a system with a low phonon density of states such as MgO. This is simple and effective, but apparently precludes the possibility of a progressive chemical optimization.

A molecular-based analogue of this construction employed a whole monolayer of terbium bis-phthalocyanine complexes TbPc_2_ on MgO/Ag(100), rather than a single Ho atom.^81^ It was reported that magnetic remanence and hysteresis opening obtained with TbPc_2_ on MgO tunnel barriers outperform the ones of any other surface adsorbed SMM as well as those of bulk TbPc_2_. However, hysteresis disappears above 8 K in this molecular monolayer, in contrast to a blocking temperature of 30 K for single Ho atoms. Whereas the phonon spectrum of MgO is equally poor in both cases, the difference might lie in the richer phonon spectrum available to a compact monolayer of TbPc_2_ molecules compared to isolated and thus “cleaner” Ho atoms.

Finally, let us discuss a last issue from the point of view of calculations. As previously stated in eqn (1), the dynamical contribution to the temperature dependence of the magnetic anisotropy consists of two parts: the contribution of acoustic phonons and that of optical phonons. Over time, the importance of including both acoustic and optical phonons in spin dynamics has been stressed.^44^,^53^,^74^–^77^,^82^ Indeed, some studies had to be revisited for not properly including the effects of both of them.^49^,^83^ Thus, any theoretical model to be developed should first consider the importance that both acoustic and optical phonons could have on spin relaxation before arbitrarily neglecting either of them.

A well-known problem which is nevertheless not routinely considered in molecular spin dynamics is the first static contribution in eqn (1), despite having been repeatedly proven to be crucial in other contexts.^48^,^53^,^74^–^77^ Recently, in the relevant experimental regime, magnetic relaxation times (extensible to spin–lattice decoherence times) are being calculated by assuming that relaxation is mainly phonon-induced.^15^,^44^ But, this static contribution can also play a key role in the thermal modulation of spin Hamiltonian parameters, so its effects should be considered in calculating these relaxation times. Moreover, such a static contribution may become important at temperatures higher than the nitrogen boiling point and thus would have to be considered in this thermal regime. Although *ab initio* calculations on spin dynamics are close to recovering the experimental order of magnitude of relaxation rates, discrepancies like temperature independent shifts between experimental and calculated relaxation times still remain.^15^ Already in 1973, it was discussed that the inclusion of the exact density of phonon states instead of the Debye *ω*^2^ value can automatically incorporate the effect of the lattice thermal distortion.^48^,^60^ Thus, if this static effect is finally proven to be important in calculating magnetic relaxation rates, its proper inclusion could be undertaken by considering the exact phonon spectrum or, alternatively, its most relevant parts.

Nowadays, the exact phonon spectrum in molecular systems is not considered in the calculations of the spin dynamics. For example, the relaxation dynamics of [Dy(Cp^ttt^)_2_]^+^ were consistent between the crystalline phase and the amorphous frozen solution. Thus, localized molecular vibrations were assumed to govern the spin dynamics, and, therefore, only the gas-phase vibrational modes were considered.^15^ In the discussion, the authors pointed out that this oversimplification was a possible cause behind the fact that the temperature dependence of their calculated Raman mechanism deviates considerably from the experiment. A second example is provided by the molecule [(tpa^Ph^)Fe]^–^. In this case, acoustic phonons were included in the modelling,^44^ but only in a minimal expression, namely the unit cell gamma-point normal modes. Again, this simplification might be behind the order-of-magnitude deviation between predicted and experimental relaxation times.

Notice that given the importance of an exact phonon dispersion in determining spin relaxation at elevated temperatures, it will be crucial to obtain insight into the environmental phonons that can contribute to these relaxation processes. Indeed, one needs to recall that phonons are an essential part of the dissipation pathway towards the thermal bath.^29^ In this pathway, local vibrations play the role of a link between spin states and phonons.^84^ Thus, the relationship between molecular vibrational modes and the lattice phonons that contribute to spin relaxation should be investigated, which can only be done if we have access to a good estimate of the phonon dispersion. This would allow us to understand which structural features of the magnetic molecules control their relation to the bulk environment and thereby govern spin relaxation.

## 4. Conclusions

The miniaturisation of classical magnetic storage and its contribution to the next generation of quantum technologies will require characterizing and blocking all relevant relaxation pathways in molecular nanodevices. For decades now, the molecular magnetism community has been dealing with the issue of spin dynamics in SMMs, but, as we have briefly reviewed here, this is still far from being a solved problem. Most of the efforts, both on the theoretical and experimental sides, have been focused on the understanding and raising of the barrier, assuming a dominating Orbach process. Indeed, the barrier has been enhanced, following simple magneto-structural correlations such as targeting a linear crystal field for oblate f-ions, but this is not the end of the story. Lately, but mainly since 2017, a new trend is rising: the trend of recognizing the vital importance of the spin–phonon coupling and thus of trying to theoretically understand this mechanism, in the hope of facilitating rationalisation and molecular design. The goal is, of course, to fabricate molecules where relaxation is blocked not only *via* a barrier but also in terms of coupling with vibrations, to impede, among others, Raman processes. This is a challenge that involves developing new theoretical tools, as well as revisiting some known ones. The first steps have already been taken in a handful of germinal papers. The key aim is to identify those vibrational modes prone to cause magnetic relaxation, with the ultimate goal of designing spin systems with long relaxation times at high temperature. Understanding these relaxation processes allows determining which atoms or groups are involved in the most detrimental vibrational modes. Then, *design rules* can be proposed to rationally synthesise robust molecular spin qubits and molecular magnets protected against vibration-induced relaxation. At this point, it is a matter of chemical ingenuity to obtain variations of these complexes where these atoms or groups have been substituted by others, which are heavier or are otherwise impeded in their movement. A perspective for the immediate future of spin–phonon investigation would be combining the strong parts of two or more of the existing theoretical approaches. Together with an experimental effort, this kind of integrative approach could finally provide some solid understanding in a field where currently the theory struggles to keep up with experimental discoveries.

## Conflicts of interest

There are no conflicts to declare.
